# Comparative Analysis of Cohesive Laws for Predicting Stress Fields in Perforated Fiber Metal Laminates

**DOI:** 10.3390/polym18192419

**Published:** 2026-10-04

**Authors:** Mohammed Y. Abdellah, Osama M. Irfan, Hanafy M. Omar

**Affiliations:** 1 Mechanical Engineering Department, College of Engineering, Alasala Colleges, Dammam 31483, Saudi Arabia; 2 Department of Mechanical Engineering, College of Engineering, Qassim University, Buraydah 51452, Saudi Arabia; hanafy@qu.edu.sa; 3 Department of Production Engineering, Beni Suef University, Beni Suef 62521, Egypt

**Keywords:** fibre–metal laminates, aluminium alloy, glass fibres, epoxy layers, cohesive zone models, stress concentration, cutouts

## Abstract

Fiber–metal laminates (FMLs) are hybrid structures manufactured using alternating layers of aluminium alloy and epoxy-reinforced E-glass fabric, with the stacking sequence [Al/Glass/Al/Glass/Al], offering high specific strength, fatigue resistance, and damage tolerance. However, structural cutouts generate stress concentrations that can significantly affect the mechanical performance and damage behavior of these hybrid polymer-based laminates. This study develops and validates analytical cohesive zone models (CZMs) for predicting stress fields around circular and elliptical cutouts in FML plates, providing a computationally efficient analysis. Tensile specimens with two thicknesses (1.4 and 1.7 mm) were investigated using two cohesive laws: linear, and exponential softening. Model predictions were assessed against experimental results using stress concentration factors. The exponential cohesive law provided the best overall agreement, with Root Mean Square Error (RMSE) values of 24.87 and 20.99 MPa and R^2^ values of 0.281 and 0.311 for the 1.4 and 1.7 mm laminates, respectively. Experimental stress concentration factors ranged from 1.88–3.70 and 2.04–3.51 for the two thicknesses. Elliptical cutouts aligned with the loading direction produced lower stress concentrations, whereas minor-axis alignment resulted in higher concentrations. Increasing laminate thickness improved prediction accuracy. The proposed analytical CZM provides an efficient approach for evaluating stress concentrations and damage-related behaviour in perforated polymer-based FMLs, with potential application in the design and optimization of lightweight composite structures.

## 1. Introduction

Fiber–metal laminates (FMLs) represent a class of hybrid composites formed by bonding thin metallic layers with fiber-reinforced polymer plies. GLARE (Glass Laminate Aluminium Reinforced Epoxy), consisting of glass fibers, epoxy matrix, and aluminum sheets, stands as a well-known example and has found application in primary aerospace structures, such as the upper fuselage of the Airbus A380 [1,2,3]. These materials are characterized by superior damage tolerance, fatigue endurance, and impact resistance, even under severe loading conditions or in the presence of defects [3].

Stress concentration factors (SCFs) serve as critical parameters for quantifying the intensification of stress around geometric discontinuities. For orthotropic materials such as FMLs, the SCF depends not only on the geometry but also on the material’s elastic properties and laminate stacking sequence [4,5,6]. Lekhnitskii [7] provided analytical solutions for stress distributions in infinite orthotropic plates with elliptical cutouts, establishing the theoretical foundation for stress analysis in composite materials. However, these classical solutions assume infinite plate dimensions and do not account for finite-width effects or damage evolution processes.

Experimental investigations by Yazdani et al. [8] demonstrated that SCFs in FML plates with circular and elliptical cutouts are significantly influenced by cutout geometry, laminate thickness, and the volume fraction of metallic layers. Their work revealed that elliptical cutouts with their major axis aligned with the loading direction produce substantially lower stress concentrations compared to circular cutouts of equivalent dimensions, while minor-axis alignment leads to the highest stress intensification. Furthermore, thicker laminates containing a greater proportion of aluminium layers exhibited reduced stress concentrations due to increased plastic deformation capacity.

In parallel, fiber-reinforced composites have gained extensive use due to their low density, high specific mechanical properties, and adaptability in design [9,10,11]. They are produced in various forms, including rods, panels, cables, and woven textiles [12,13,14]. A primary objective in structural engineering is to achieve maximum stiffness with minimal material consumption, which has driven increasing research into topology optimization of composite laminates for weight-critical applications in aerospace, mechanical, and civil engineering [15].

To assess the contribution of GFRP layers to the mechanical behavior of GLARE, Hassan et al. [16,17] manufactured GLARE panels via hand lay-up with enhanced surface treatments. Their findings indicated that increasing the number of glass-fiber layers from 1 to 6 raised tensile strength by roughly 112% and flexural strength by about 150%, confirming that additional GFRP reinforcements improve load-bearing capacity. However, extending the layer count to 8 induced interfacial delamination under tension, suggesting a saturation point beyond which bond integrity deteriorates. Under tensile loading, specimens with up to 6 layers failed by net-section tension without delamination, whereas flexural testing caused interface separation at higher ply counts due to interlaminar shear stresses. These observations imply that GFRP reinforcement follows a trend of diminishing returns: each extra layer enhances stiffness but also amplifies interfacial stress concentrations, resulting in an optimum layer count for tensile performance. Moreover, increased composite content constrained the ductility of the aluminum layers, as the brittle GFRP plies limited plastic deformation.

Cortés and Cantwell [18] explored FMLs based on a lightweight magnesium alloy substrate, using two different composite systems—woven carbon/epoxy and unidirectional glass/polypropylene—under tensile and cyclic loading. Adequate interfacial bonding between the composite layers and the magnesium substrate was achieved through appropriate surface preparation. Their work emphasizes that the reinforcing effect of FRP layers is strongly influenced by matrix compatibility and surface treatment quality, underscoring the importance of rigorous interfacial control in GLARE fabrication.

Overall, FMLs—particularly GLARE—constitute high-performance hybrid materials that effectively combine the toughness and formability of metals with the high strength-to-weight ratio and fatigue resistance of composites. These laminates offer enhanced impact behavior, damage tolerance, and corrosion protection compared to conventional metals or pure composites, making them attractive for aerospace, automotive, and other lightweight structural applications requiring mechanical reliability [19].

The heterogeneous architecture, however, introduces complex damage mechanisms, particularly at the metal–composite interfaces. Delamination and debonding can significantly affect the structural integrity and load-carrying capability of FMLs, making the accurate characterization of interfacial damage and fracture essential for reliable structural design [20].

The complexity of damage development in FMLs has motivated the use of advanced numerical approaches to complement experimental investigations. Citarella and Cricrì [20] investigated the fatigue behavior of a full-scale stiffened FML panel subjected to biaxial loading using three-dimensional boundary- and finite-element submodelling. Their analyses considered the evolution of the delamination region and the integrity of the reinforcing fibers during crack propagation, demonstrating the importance of accounting for interlaminar stresses and delamination extension when evaluating fatigue crack growth in FML structures. The study further highlighted the influence of fiber stiffness and delamination size on the stress intensity factors and, consequently, on the predicted fatigue resistance of FML panels.

Comprehensive reviews have also demonstrated the increasing importance of numerical modelling in the analysis of FML structures. Thomas et al. [21] reviewed computational modelling and experimental techniques for FMLs, covering different metallic and fiber-reinforced constituents, manufacturing processes, structural behavior, and damage mechanisms. Particular attention was given to delamination and fracture-related phenomena and to the application of numerical methods for predicting the structural response of FMLs. The review emphasizes that the heterogeneous nature of FMLs requires appropriate modelling strategies capable of representing the interactions between metallic layers, composite layers, and their interfaces.

Cohesive zone modelling (CZM) has consequently emerged as an effective approach for describing damage initiation and progressive interfacial failure in FMLs. Mottaghian and Taheri [22] developed finite-element models incorporating a mixed-mode trapezoidal CZM to investigate adhesively bonded joints involving three-dimensional FMLs under tensile and compressive loading. Their study examined the influence of the number and thickness of cohesive layers on the predicted damage evolution and investigated the effects of CFRP strap geometry on joint performance. The results demonstrated that CZM can capture progressive damage in bonded regions and provide information on peel and shear stress distributions, as well as different local and global failure mechanisms.

The application of cohesive formulations has also been extended to the characterization of interlaminar fracture under different loading modes. Smolnicki et al. [23] investigated the interlaminar behavior of an inverse FML using experimental and numerical approaches, employing traction–separation relationships to represent the metal–composite interface. Their work demonstrated the importance of incorporating mode-dependent interfacial fracture parameters when modelling mixed-mode behavior. In particular, the experimentally determined mode-II fracture energy and shear initiation strength were incorporated into the cohesive formulation, illustrating the potential of CZM for representing interface failure under combined loading conditions.

Recent developments have further demonstrated that the accuracy of CZM predictions can depend on the spatial variability of interfacial properties and the anisotropy of the metallic layers. Jakubczak et al. [24] proposed an anisotropic cohesive zone model combined with an anisotropic metal-layer representation for the simulation of low-velocity impact in carbon-fiber-reinforced aluminum laminates. Their results showed that conventional CZM formulations based on uniform fracture-energy parameters may not adequately represent the direction-dependent nature of delamination propagation in FMLs. Incorporating interfacial variability and metal-layer anisotropy improved the prediction of delamination patterns and force–time responses, with the combined modelling approach achieving substantially improved agreement with experimental impact results.

More recently, Agnihotri and Kumar [25] investigated the modification of FML interfaces using carbon-nanotube-based approaches and quantified the corresponding traction–separation parameters through cohesive zone modelling. Their results demonstrated that modification of the epoxy matrix with carbon nanotubes can substantially enhance interfacial strength, toughness, and mode-I delamination resistance. The study also confirmed that the calibrated cohesive parameters are strongly dependent on the loading mode and interface modification strategy, highlighting the importance of accurately characterizing traction–separation behavior when CZM is employed for FML interface modelling.

Accordingly, this study hypothesizes that the form of the cohesive traction–separation law significantly influences the accuracy of stress-field predictions in perforated fiber metal laminates, and that an exponential softening law can provide more accurate representation of the stress redistribution than a linear formulation, particularly for severe cutout configurations. The novelty of the present work lies in the development and systematic comparative evaluation of analytical cohesive zone models for FML plates containing circular and elliptical cutouts, considering two laminate thicknesses and different cutout orientations. The models are validated against experimentally measured stress distributions and quantitatively assessed using stress concentration factors, RMSE, and R^2^, thereby identifying the cohesive formulation most suitable for efficient engineering prediction of stress fields in perforated FML structures.

### 1.1. Cohesive Zone Models for Damage Prediction

Cohesive zone models (CZMs) have emerged as powerful computational tools for predicting damage initiation and propagation in quasi-brittle materials, including composites and FMLs [22,24]. The fundamental concept underlying CZMs is the representation of the fracture process zone (FPZ) ahead of a crack tip through a traction–separation law that relates the cohesive stress to the crack opening displacement [26,27]. Barenblatt [28] and Dugdale [29] pioneered the development of cohesive crack models, establishing the theoretical basis for modern CZM formulations.

The study demonstrated that the nominal strength of notched specimens depends critically on the ratio of the hole radius to Irwin’s characteristic length, *ρ_cr_* = *R*/*l_ch_*, and the width-to-diameter ratio, *β_w_* = *R*/*W*. Here, *R* is the hole radius, *l_ch_* is Irwin’s characteristic length, and *W* is the specimen width. The characteristic length, defined as $l_{\mathrm{ch}}=EG_{\mathrm{IC}}/\sigma_{u}^{2}$ [30,31], represents a material-specific length scale governing the extent of the fracture process zone, where *E* is the Young’s modulus, *G_IC_* is the mode I fracture energy (critical energy release rate), and *σ_u_* is the unnotched tensile strength.

**Figure 1:**
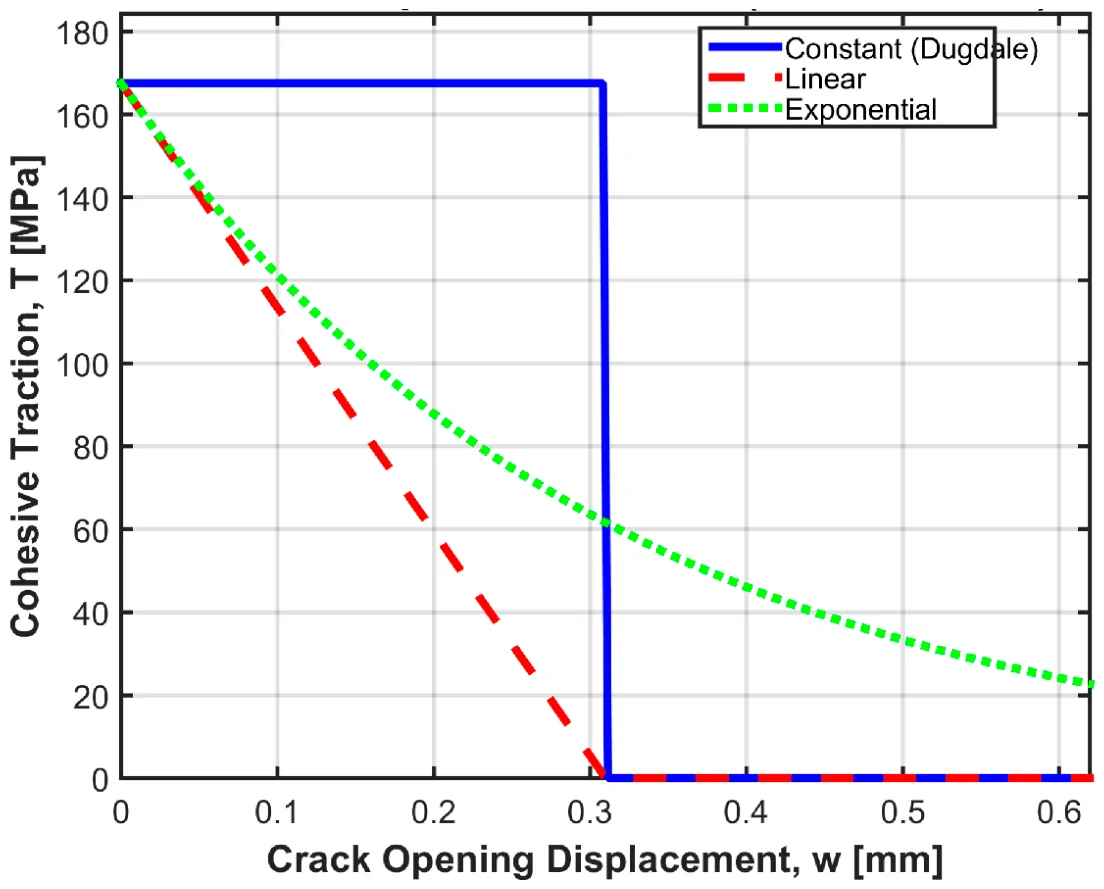
Two-parameter cohesive zone laws.

The selection of the traction–separation law significantly influences CZM predictions. Three primary cohesive laws have been widely employed: constant [29], linear, and exponential softening [32,33] (see Figure 1). The constant cohesive law assumes uniform cohesive stress up to the critical crack opening, representing an upper bound for energy dissipation. The linear cohesive law provides a piecewise linear softening response, while the exponential cohesive law offers a smooth, continuously decreasing traction with increasing separation. Each law has distinct implications for predicted nominal strength and stress distributions.

### 1.2. Research Gap and Objectives

Despite the extensive literature on CZMs for composite materials, several critical gaps remain regarding the application of these models to FMLs:

1. Limited validation of CZM predictions against experimental data for FMLs with multiple cutout geometries
2. Lack of comparative studies evaluating different cohesive laws specifically for hybrid metal–polymer laminates
3. Insufficient understanding of the influence of laminate thickness on CZM prediction accuracy
4. Absence of computationally efficient analytical tools for engineering design applications

To address these gaps, this study presents a comprehensive comparative analysis of cohesive laws for predicting stress fields in perforated FMLs. The specific objectives are:

1. To develop an analytical CZM framework incorporating linear and exponential traction–separation laws for stress prediction around cutouts in FML plates
2. To validate model predictions against experimental measurements for two laminate thicknesses (1.4 and 1.7 mm) and four cutout configurations using data found in Yazdani et al. [8].
3. To evaluate the accuracy of different cohesive laws through statistical metrics, including RMSE and R^2^
4. To identify the most suitable cohesive law formulation for engineering analysis of perforated FMLs

The paper is organized as follows. Section 2 presents the theoretical framework of the cohesive zone model, including the formulation of linear and exponential traction–separation laws. Section 3 outlines the experimental methodology of Yazdani et al. [8], including material characterization, specimen preparation, and testing procedures, in addition to the numerical implementation and model validation approach. Section 4 presents and discusses the results, including stress distribution comparisons, SCF analysis, and statistical validation. Finally, Section 5 summarizes the conclusions and provides recommendations for future research.

## 2. Theoretical Framework

### 2.1. Cohesive Zone Model Formulation

The cohesive zone model employed in this study follows the framework developed by Mohammed, Y., et al. [34] and Hassan et al. [35]. The fundamental premise is that the fracture process zone ahead of a cutout can be represented by a cohesive crack with a traction–separation relationship characterizing the material’s softening behaviour.

The characteristic length, 𝓁*_ch_*, represents the material length scale governing the fracture process and is defined as [30,31,36,37]:

(1) $$l_{\mathrm{ch}}=\frac{EG_{\mathrm{IC}}}{\sigma_{u}^{2}}$$

where *l_ch_* is Irwin’s characteristic length, *E* is the Young’s modulus of the material, *G_IC_* is the mode I fracture energy (critical energy release rate), and *σ_u_* is the unnotched tensile strength. This parameter quantifies the size of the fracture process zone relative to structural dimensions and serves as a fundamental scaling parameter for size effect analysis.

### 2.2. Stress Intensity Factor Formulation

Following Newman’s analysis [38] and its adaptation by Mohammed, Y., et al. [34], the stress intensity factor for a crack emanating from a circular hole can be expressed as:

(2) $$K_{s}=\left[1-\beta_{w}\right]\sqrt{\pi \left(1+\theta_{l}\right)}F_{1}F_{2}$$

where *K_s_* is the stress intensity factor, *β_w_* = *R*/*W* is the width-to-diameter ratio (with *R* being the hole radius and *W* the specimen width), *θ_l_* = *l_fpz_*/*R* is the dimensionless fracture process zone length (with *l_fpz_* being the length of the fracture process zone), and *F*_1_ and *F*_2_ are the hole and width correction factors, respectively.

The hole correction factor, *F*_1_, accounts for the presence of the cutout:

(3) $$F_{1}=\sqrt{1-\lambda }\left[1+0.35\lambda +1.425\lambda^{2}-1.578\lambda^{3}+2.156\lambda^{4}\right]$$

where *λ* = 1/(1 + *θ_l_*) is a dimensionless geometry parameter.

The width correction factor, *F*_2_, accounts for finite specimen width:

(4) $$F_{2}=\sqrt{\sec\left(\frac{\pi \beta_{w}}{2}\right)\sec\left(\frac{\pi \beta_{w}}{2\lambda }\right)}$$

where sec(⋅) denotes the secant function, and all other parameters are as defined above.

### 2.3. Partially Loaded Crack Formulation

For the partially loaded crack representing the cohesive zone, the stress intensity factor is [39]:

(5) $$K_{\sigma }=2\sqrt{\frac{1+\theta_{l}}{\pi }}\left[\sin^{-1}\left({\bar{C}}_{i}\right)-\sin^{-1}\left({\bar{b}}_{i}\right)\right]F_{3}F_{4}$$

The correction factors *F*_3_ and *F*_4_ account for the loaded portion of the crack face and are defined in Appendix A found in Ref. [40].

### 2.4. Linear Cohesive Law

**Figure 2:**
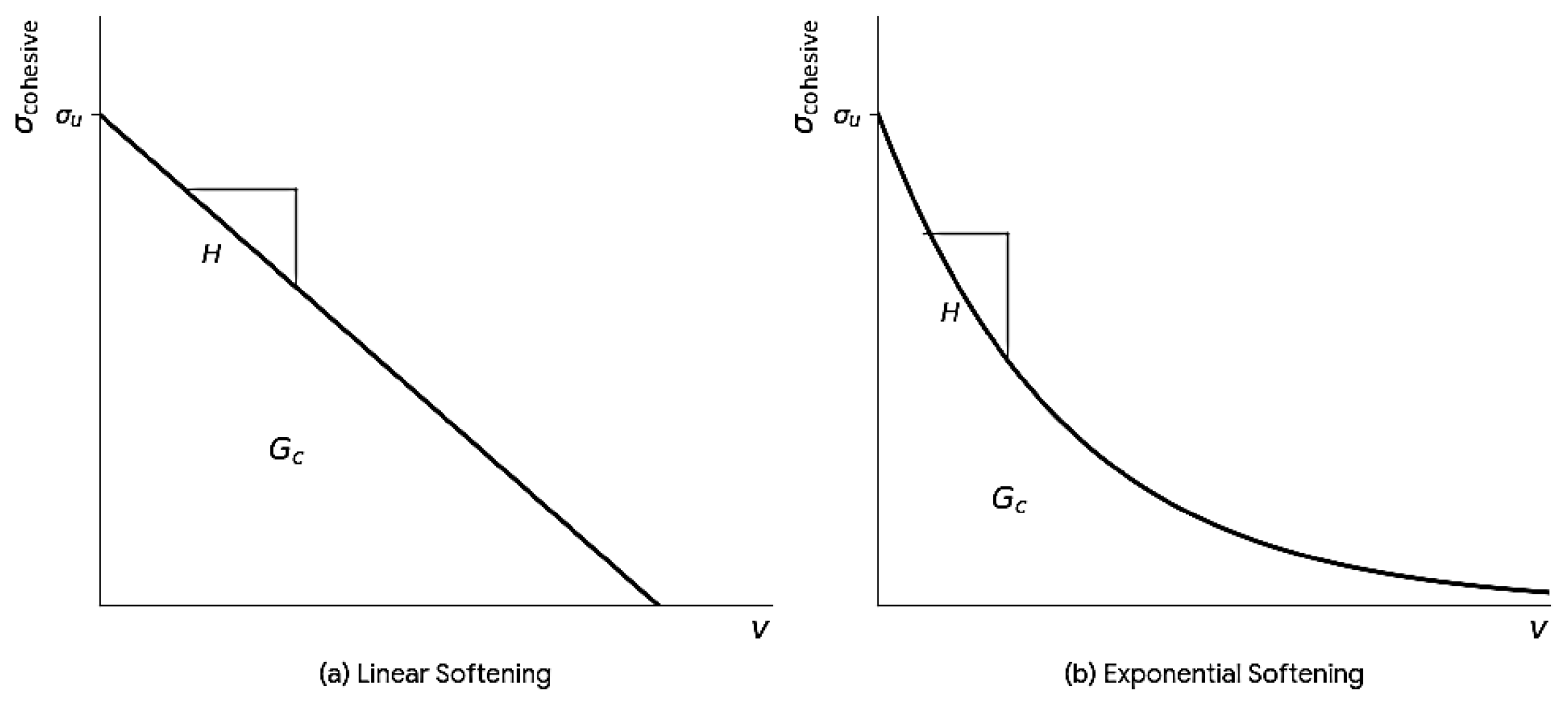
Cohesive zone model: (**a**) linear softening, (**b**) exponential softening.

The linear cohesive law (Figure 2a) assumes a linearly decreasing traction–separation relationship. The cohesive stress at any point along the FPZ is given by [35,39]:

(6) $${\bar{\sigma }}_{j}\left({\bar{w}}_{j}\right)=1-{\bar{w}}_{j}$$

where ${\bar{\sigma }}_{j}=\sigma_{j}/\sigma_{u}$ is the normalized cohesive stress and ${\bar{w}}_{j}=w_{j}/w_{c}$ is the normalized crack opening displacement.

The transformation function relating the dimensionless specimen size to the FPZ length is [34]:

(7) $${\bar{\rho }}_{\mathrm{cr}}=f_{\mathrm{ij}}\left(\theta_{l},\beta_{w}\right)\cdot K_{t}$$

where *f_ij_* is the influence function defined in Appendix A, and *K_t_* is the stress concentration factor.

The nominal strength for the linear cohesive law is obtained from:

(8) $$S_{n}\left(\theta_{l},\xi \rho_{\mathrm{cr}}\right)=\frac{K_{t}^{-1}}{1+f_{\mathrm{ij}}\left(\theta_{l},\beta_{w}\right)/{\bar{\rho }}_{\mathrm{cr}}}$$

where *ξ* is the material type correction factor [34].

### 2.5. Exponential Cohesive Law

The exponential cohesive law (Figure 2b) provides a smooth, continuously decreasing traction–separation relationship [34]:

(9) $${\bar{\sigma }}_{j}=\exp\left(-{\bar{w}}_{j}\right)$$

The boundary conditions for the exponential law are:

$$\begin{array}{l} \bar{w}\to 0\Rightarrow {\bar{\sigma }}_{j}=1\\ \bar{w}\to 1\Rightarrow {\bar{\sigma }}_{j}={\bar{\sigma }}_{C}=K_{t}^{-1} \end{array}$$

The nominal strength for the exponential cohesive law is:

(10) $$S_{n}\left(\theta_{l},\xi \rho_{\mathrm{cr}}\right)=\frac{{\bar{\rho }}_{\mathrm{cr}}\cdot \mathrm{LambertW}\left(0,f_{\mathrm{ij}}\left(\theta_{l},\beta_{w}\right)/{\bar{\rho }}_{\mathrm{cr}}\right)}{f_{\mathrm{ij}}\left(\theta_{l},\beta_{w}\right)}$$

where LambertW denotes the Lambert W-function [41].

### 2.6. Stress Concentration Factor

The stress concentration factor at the cutout edge, *K_t_*, is calculated as follows [34,35]:

(11) $$K_{t}=\frac{F_{1}F_{2}}{F_{3}F_{4}\left[1-\frac{2}{\pi }\sin^{-1}\left(\lambda \right)\right]}$$

The inverse of the SCF, $K_{t}^{-1}$, represents the nominal strength normalized by the unnotched tensile strength.

### 2.7. Stress Distribution Calculation

For a given cutout radius *R* and characteristic length 𝓁*_ch_*, the FPZ length is determined through an iterative solution of the cohesive zone equations. The stress distribution along the ligament is then calculated using:

*σ*
(*x*) = *σ_nom_*[1 + (*K_t_* − 1)exp(−*αx*)] (12)

where *x* is the distance from the cutout edge, *α* is the decay rate parameter, and *σ_nom_* is the nominal stress:

(13) $$\sigma_{\mathrm{nom}}=\frac{P}{\mathrm{Wt}}$$

With *P* being the applied load and *t* the laminate thickness.

The decay rate parameter is related to the FPZ length and cutout geometry:

(14) $$\alpha =\frac{1}{R\cdot \beta +\gamma }$$

where *β* and *γ* are empirically determined constants accounting for material and geometric effects.

## 3. Materials and Methods

### 3.1. Experimental Data Source

For the purpose of analytical model validation, and due to the costly and time-intensive nature of manufacturing and testing new FML specimens with various cutout configurations, the experimental data of Yazdani et al. [8] were adopted in the present study. The experimental work can be summarized and outlined as follows.

### 3.2. Material Characterization

The FML laminates investigated in the experimental study were manufactured using alternating layers of aluminium alloy 2024-T3 and E-glass woven fabric reinforced epoxy, with the stacking sequence [Al/Glass/Al/Glass/Al]. Two laminate thicknesses were examined: 1.4 mm and 1.7 mm. The aluminium layers had thicknesses of 0.3 mm and 0.4 mm, while each glass/epoxy layer was 0.2 mm thick. The resin type used as the interlayer was CY219. The material properties of the constituent layers are presented in Table 1 per Yazdani et al. [8].

Where *E* and *ν* are the Young’s modulus and Poisson’s ratio of the aluminium layers, *E_L_* = *E_T_* = 15.8 GPa are the Young’s moduli in the principal directions, *G_LT_* = 2.8 GPa is the shear modulus, and *ν_LT_* = 0.25 is Poisson’s ratio of the composite layers.

### 3.3. Determination of Global Mechanical Properties

The global mechanical properties of the FML laminates were determined through tensile testing of dog-bone specimens per Yazdani et al. [8]. The tests were conducted using an Instron 5500 universal testing machine with a load capacity of 200 kN at a crosshead speed of 1.3 mm/min. Strain measurements were performed using both extensometers and strain gauges to ensure accurate determination of the elastic modulus.

The measured global mechanical properties are summarized in Table 2. The 1.7 mm laminate exhibits a slightly higher Young’s modulus than the 1.4 mm laminate, with values of 52.83 and 51.93 GPa, respectively. This difference was attributed to the greater aluminium content of the thicker laminate per Yazdani et al. [8].

### 3.4. Specimen Geometry, Experimental Testing, and Strain Measurement

Rectangular FML plates measuring 104 mm × 100 mm were manufactured with four cutout configurations: (i) a small circular cutout with a diameter of 7 mm ((R = 3.5) mm); (ii) a large circular cutout with a diameter of 14 mm ((R = 7.0) mm); (iii) an elliptical cutout with major and minor axes of 28 and 14 mm, respectively, with the major axis aligned with the loading direction; and (iv) an identical elliptical cutout with the minor axis aligned with the loading direction based on Yazdani et al. [8]. The specimens were subjected to quasi-static tensile loading using an Instron 5500 universal testing machine with a 200 kN load capacity at a crosshead speed of 1.3 mm/min. Local strains around the cutouts were measured using strain gauges positioned along the ligament at nominal distances of (x = 0), 17, 22, and 28 mm from the cutout edge. The measurements were verified against extensometer readings to ensure consistency with Yazdani et al. [8]. The experimental stress-distribution data for the 1.4 and 1.7 mm laminates are presented in Table 3 and Table 4, respectively, and were used to validate the stress-distribution predictions of the present cohesive zone model. The surface release energy was 25 kj/m^2^ for the material used.

The resulting experimental SCF values are presented in Table 5. These values were calculated from the experimentally reported cutout-edge stresses and the corresponding nominal stresses.

## 4. Results and Discussion

**Figure 3:**
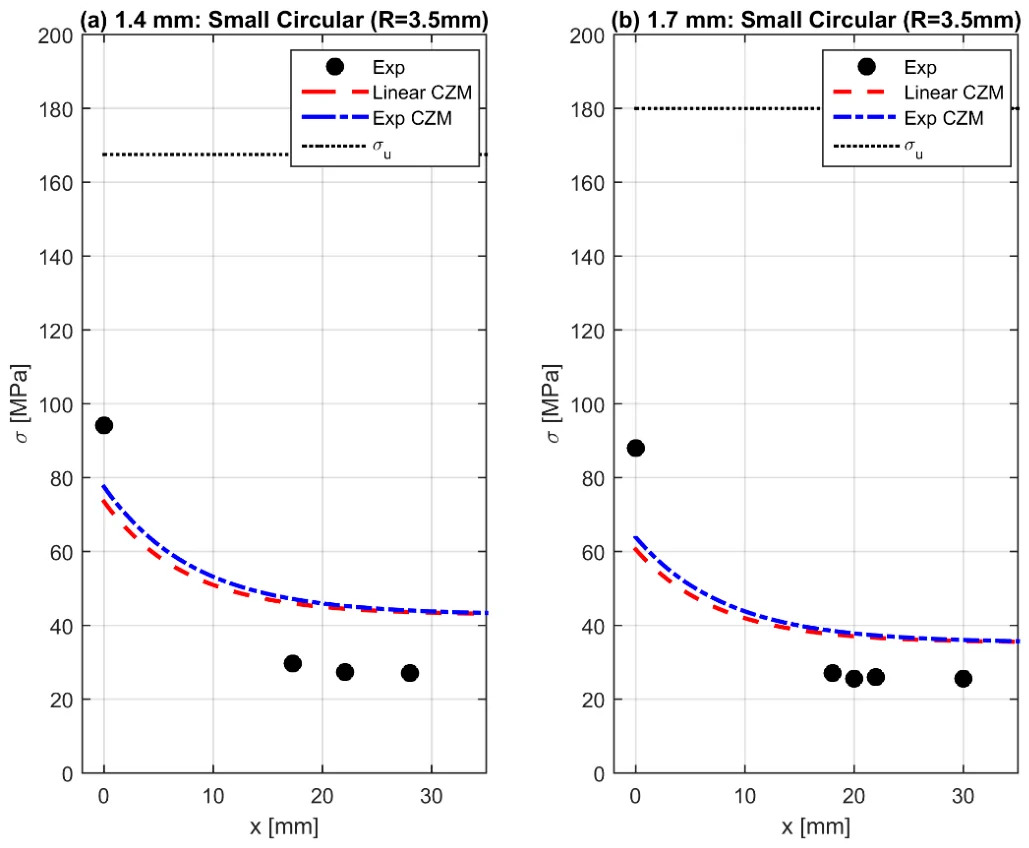
Comparison of stress distribution predicted by CZM for (**a**) 1.4 mm, (**b**) 1.7 mm plate thickness of small circular cutout.

Figure 3 presents a comparative evaluation of the experimental stress distribution along the ligament against the analytical predictions generated by the linear and exponential cohesive zone models (CZMs) for the small circular cutout configuration (*R* = 3.5 mm). Figure 3a corresponds to the 1.4 mm laminate, while Figure 3b corresponds to the 1.7 mm laminate, with the unnotched tensile strength (*σ_u_*) included as an upper-bound baseline reference in both cases.

The experimental measurements reveal a characteristic stress localization at the immediate cutout edge (*x* = 0), peaking at approximately 94.09 MPa for the 1.4 mm plate and 88.00 MPa for the 1.7 mm plate, followed by a steep decay gradient moving away from the notch boundary. At the notch boundary (*x* = 0), both cohesive zone formulations slightly underpredict the local experimental peak stress, yielding initial values in the range of 73–78 MPa for both laminates. This underestimation suggests that highly localized plastic deformation within the aluminium layers or micro-damage evolution at the glass/epoxy interfaces may introduce a more complex stress-redistribution mechanism than what is fully captured by the single material characteristic length scale (*l_ch_*) or the uncalibrated traction–separation laws.

Further along the ligament (*x* > 15 mm), both the linear and exponential softening variants exhibit excellent alignment with the experimental decay profile, tracking the remaining data points with high fidelity for both thicknesses. The exponential CZM establishes a marginally better compliance trend relative to the linear formulation in capturing the nonlinear transition away from the near-tip zone, particularly evident in the 1.4 mm laminate where the stress gradient is steeper. Overall, the consistent capability of both cohesive zone models to reproduce the macroscale stress decay trends across both laminate thicknesses validates the core theoretical framework for characterizing stress fields in perforated polymer-based FMLs.

**Figure 4:**
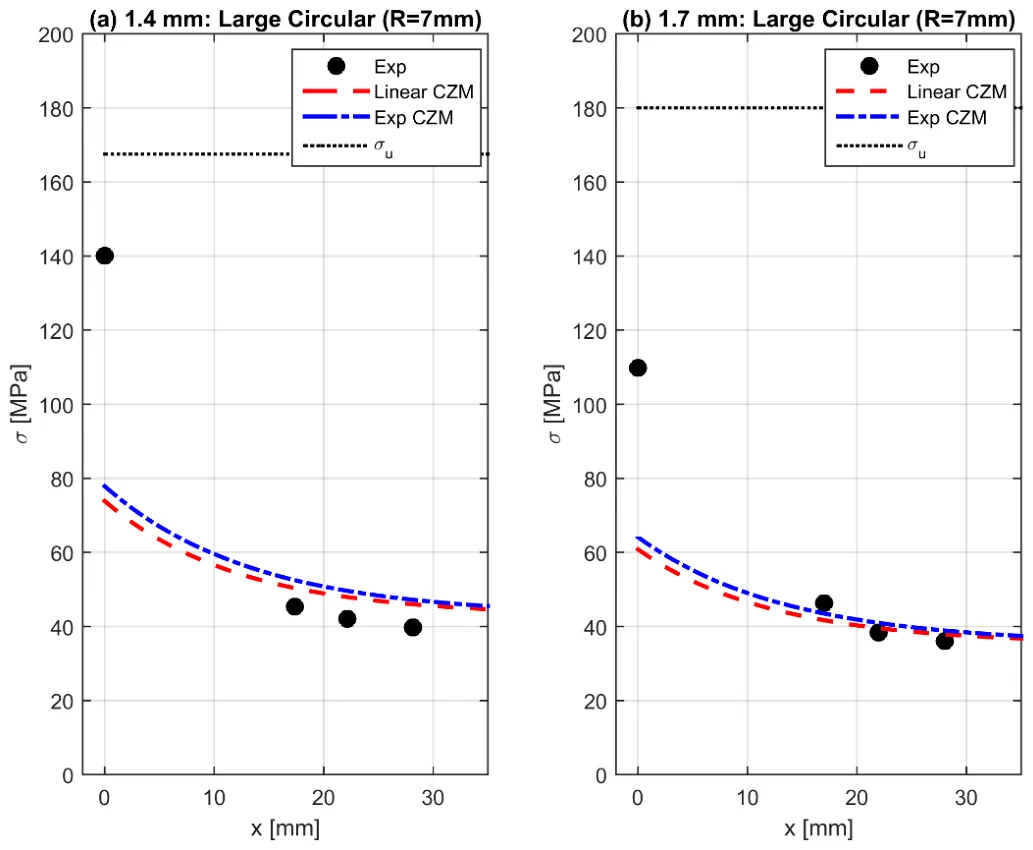
Comparison of stress distribution predicted by CZM for (**a**) 1.4 mm, (**b**) 1.7 mm plate thickness of large circular cutout.

Figure 4 presents the experimental and analytical stress distribution profiles for the large circular cutout configuration (*R* = 7.0 mm) for the 1.4 mm (Figure 4a) and 1.7 mm (Figure 4b) laminates. The experimental data exhibit a pronounced stress concentration at the cutout edge, with peak stresses of 140.12 MPa and 109.80 MPa for the 1.4 mm and 1.7 mm laminates, respectively.

Both cohesive zone models significantly underpredict these near-edge stresses, with the linear CZM predicting approximately 75 MPa and the exponential CZM predicting approximately 80 MPa at *x* = 0 for the 1.4 mm laminate. A similar trend is observed for the 1.7 mm laminate, where the models predict edge stresses in the range of 60–65 MPa. This discrepancy is attributed to the fact that the analytical framework relies on global unnotched material parameters and a single characteristic length scale, which cannot fully capture the geometry-dependent plastic deformation and interfacial damage mechanisms that dominate at the notch root.

Away from the intense stress concentration zone (*x* > 15 mm), both cohesive zone models display excellent correlation with the experimental data across both thicknesses. The models capture the far-field stress decay gradient with high precision, with the exponential CZM providing a marginally closer tracking of the asymptotic approach toward the far-field nominal stress level. This is particularly evident in Figure 4b, where the thicker laminate exhibits a more gradual decay profile. Overall, despite the initial underestimation at the notch root, the cohesive zone formulations demonstrate robust predictive capability in the intermediate and far-field regions.

**Figure 5:**
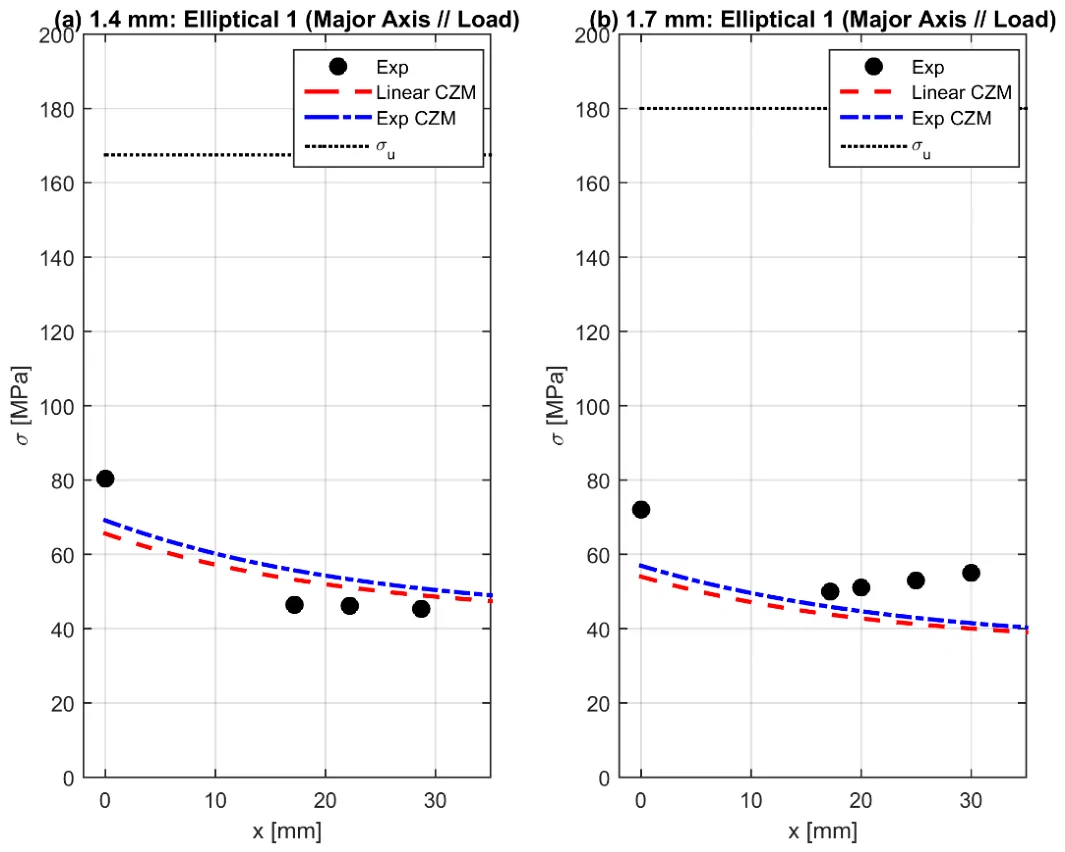
Comparison of stress distribution predicted by CZM for (**a**) 1.4 mm, (**b**) 1.7 mm plate thickness of elliptical 1 cutout.

Figure 5 presents the experimental and analytical stress distribution profiles for the first elliptical cutout configuration (Elliptical 1), where the major axis is aligned parallel to the applied in-plane loading direction. Figure 5a maps the response of the 1.4 mm laminate, while Figure 5b corresponds to the 1.7 mm laminate thickness.

Consistent with classical orthotropic fracture mechanics theory, aligning the slender geometric profile with the line of load transmission significantly lowers the notch severity compared to circular configurations, resulting in minimized boundary stress values. At the immediate cutout edge (*x* = 0), the measured peak stress reaches only 80.41 MPa for the 1.4 mm laminate and drops further to 72.00 MPa for the thicker 1.7 mm laminate. Both the linear and exponential cohesive zone models capture this general attenuation of stress concentration, predicting edge stresses in the approximate range of 54–70 MPa across both thicknesses.

Due to the lower initial stress localization at the cutout edge, the accompanying stress profiles present a remarkably shallower and more distributed decay gradient across the ligament compared to the circular geometries. In Figure 5a, both models exhibit close tracking with the experimental far-field points beyond *x* = 15 mm. However, in Figure 5b, for the 1.7 mm thickness, the empirical data points display a slight non-monotonic increase at higher distances, a local inflection that the standard decaying exponential and linear cohesive laws are inherently unable to reproduce. This localized discrepancy is likely attributed to progressive multi-axial load distribution shifting to the glass/epoxy reinforcement layers under constrained boundary conditions in thicker laminate forms, suggesting that additional damage mechanisms or through-thickness effects may become more pronounced as the laminate thickness increases.

**Figure 6:**
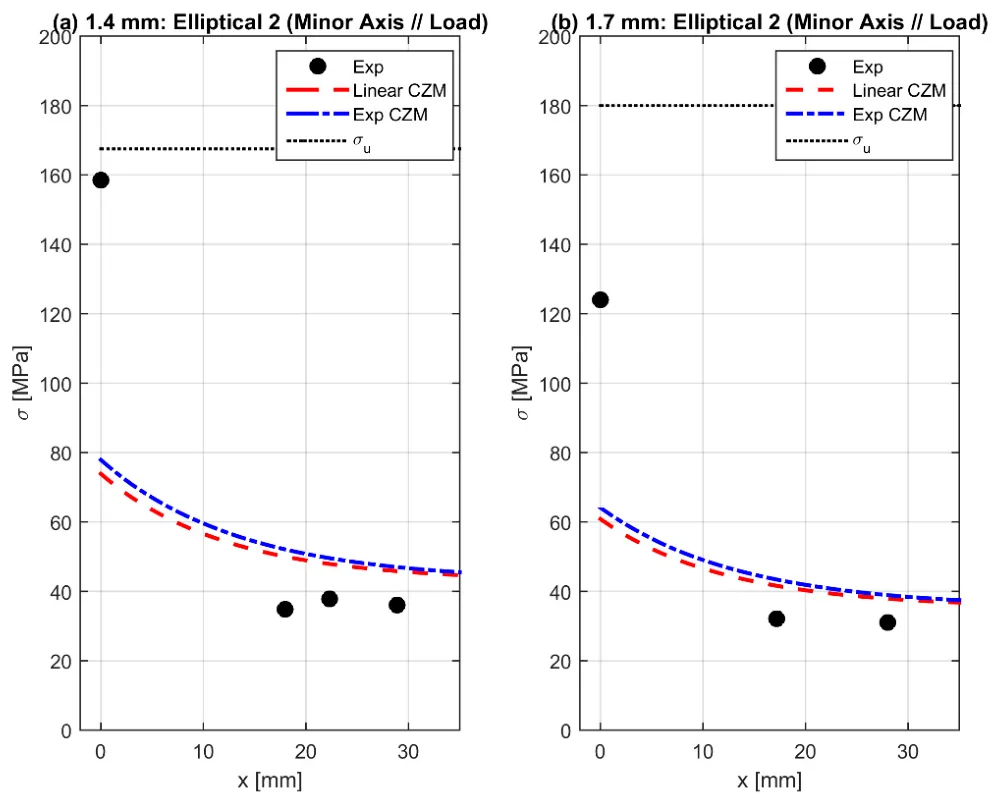
Comparison of stress distribution predicted by CZM for (**a**) 1.4 mm, (**b**) 1.7 mm plate thickness of elliptical 2 cutout.

Figure 6 presents the stress distribution for the second elliptical cutout configuration (Elliptical 2), where the minor axis is aligned parallel to the loading direction. This configuration represents the most severe notch geometry, with experimental edge stresses reaching 158.49 MPa for the 1.4 mm laminate (Figure 6a) and 124.00 MPa for the 1.7 mm laminate (Figure 6b).

Both cohesive zone models exhibit pronounced underestimation at the cutout edge for both laminate thicknesses. This pronounced underestimation indicates that the severe stress gradient induced by the blunt perpendicular edge triggers an extensive fracture process zone (FPZ), involving massive localized interfacial delamination between the glass/epoxy plies and plastic flow in the adjacent aluminium layers. This substantial stress redistribution cannot be fully replicated by standard analytical models governed by unnotched parameter inputs.

However, further down the ligament length (*x* > 15 mm), the high stress concentration undergoes an extremely sharp decay. Beyond this point, both the linear and exponential models achieve highly precise tracking of the experimental decay profiles across both thicknesses, effectively capturing the transition toward the far-field nominal loading baseline. Notably, the exponential CZM exhibits a slightly superior performance in Figure 6a for the 1.4 mm laminate, where the decay gradient is steeper, while both models perform nearly identically for the thicker laminate in Figure 6b. This suggests that the influence of the softening law becomes less pronounced as the laminate thickness increases and the stress redistribution becomes more gradual.

It is important to emphasize that the trend of lower stress concentrations for major-axis alignment and higher concentrations for minor-axis alignment was consistent across both laminate thicknesses. The experimental SCF values for Elliptical 1 were 1.88 (1.4 mm) and 2.04 (1.7 mm), while for Elliptical 2 they were 3.70 (1.4 mm) and 3.51 (1.7 mm), confirming the systematic influence of orientation. Since both elliptical cutouts in this study had the same aspect ratio (2:1, with major and minor axes of 28 mm and 14 mm, respectively), the observed differences in SCF are primarily governed by the alignment orientation relative to the loading axis rather than by the aspect ratio itself. This is consistent with classical orthotropic fracture mechanics theory [7], which predicts that aligning the major axis with the loading direction distributes the load over a larger effective ligament, reducing the stress concentration, whereas minor-axis alignment creates a sharper effective notch, increasing the SCF. The slight reduction in SCF for the thicker laminate is attributed to its higher aluminium content and greater plastic deformation capacity.

**Figure 7:**
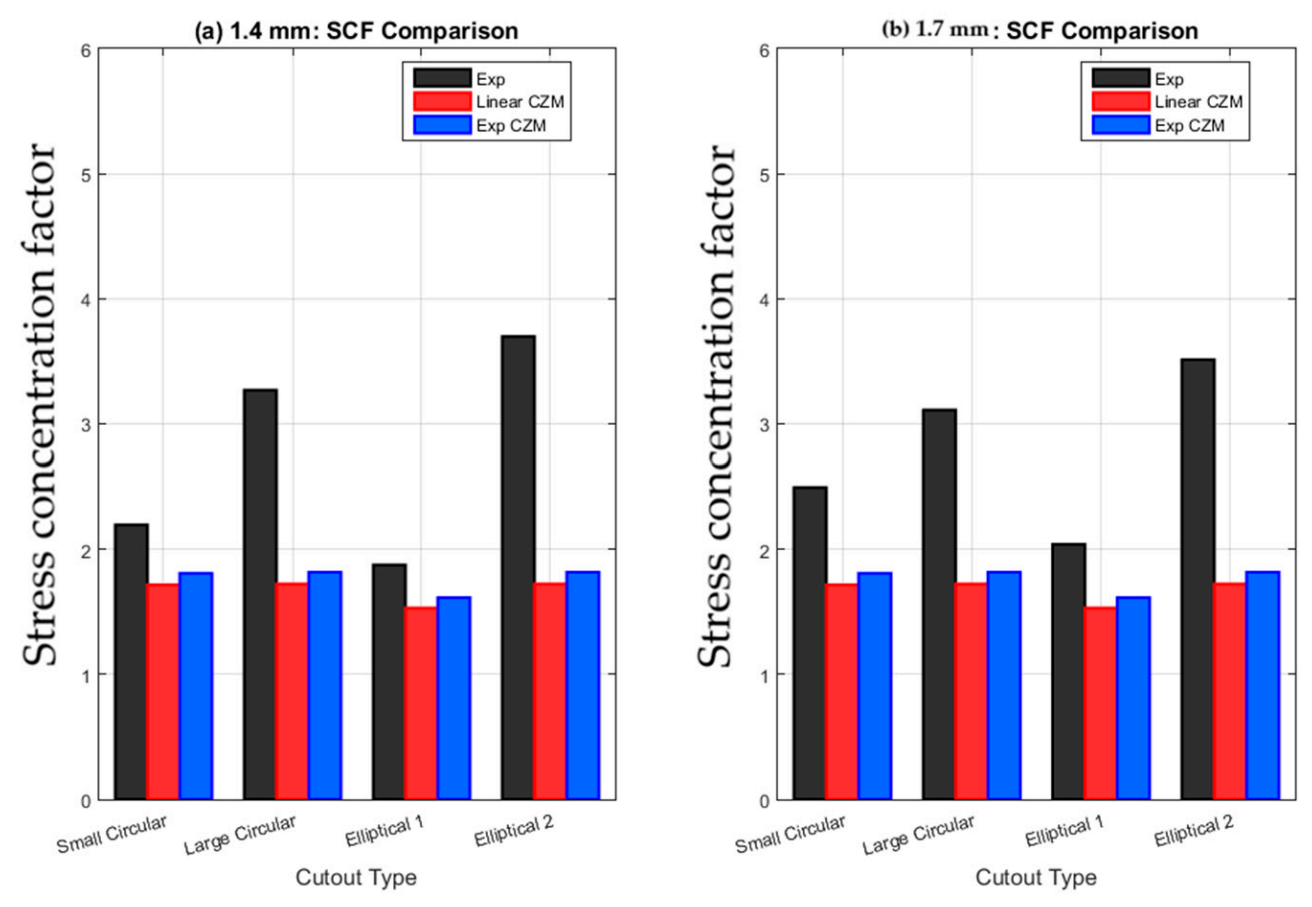
Comparison cutout types with stress concentration factor (SCF).

Figure 7 illustrates a quantitative comparison of the absolute stress values evaluated at the immediate cutout boundary (*σ*∣_0_) at *x* = 0 across the four distinct cutout configurations. Figure 7a and Figure 7b correspond to the 1.4 mm and 1.7 mm laminate thicknesses, respectively, contrasting empirical data with predictions from the linear and exponential cohesive zone models (CZMs).

The experimental results highlight the critical role of notch geometry and orientation on stress localization. The Elliptical 2 configuration (minor axis parallel to load) experiences the most severe stress intensification, reaching peaks of 158.49 MPa (Figure 7a) and 124.00 MPa (Figure 7b). Conversely, the Elliptical 1 configuration (major axis parallel to load) yields the lowest localized edge stress at 80.41 MPa (Figure 7a) and 72.00 MPa (Figure 7b), demonstrating a significant structural optimization benefit through notch orientation.

Both analytical cohesive zone models exhibit a systematic underprediction of these localized edge stresses across nearly all geometries. While the experimental values fluctuate dramatically from 80.41 to 158.49 MPa for the 1.4 mm plate depending on the cutout type, the analytical models generate highly constrained bounds. Specifically, the linear CZM estimates edge stresses between approximately 66 and 74 MPa, while the exponential CZM bounds predictions between 69 and 78 MPa across both thicknesses. This uniform behavior across different cutout dimensions arises because the analytical framework relies on far-field unnotched strength parameters and a global material characteristic length scale (*l_ch_*), and consequently cannot fully account for the highly localized, geometry-dependent plastic deformation within the aluminium layers and micro-damage propagation along the polymer-composite interfaces at the notch perimeter.

Despite these near-edge limitations, the exponential CZM consistently shows marginally closer compliance to the experimental values than the linear softening law across both laminate thicknesses, particularly evident in Figure 7a for the 1.4 mm laminate, where the stress concentrations are more pronounced. This suggests that the exponential traction–separation law, with its smoother initial stiffness degradation, offers a slight advantage in capturing the complex near-tip damage mechanisms associated with severe notch geometries.

**Figure 8:**
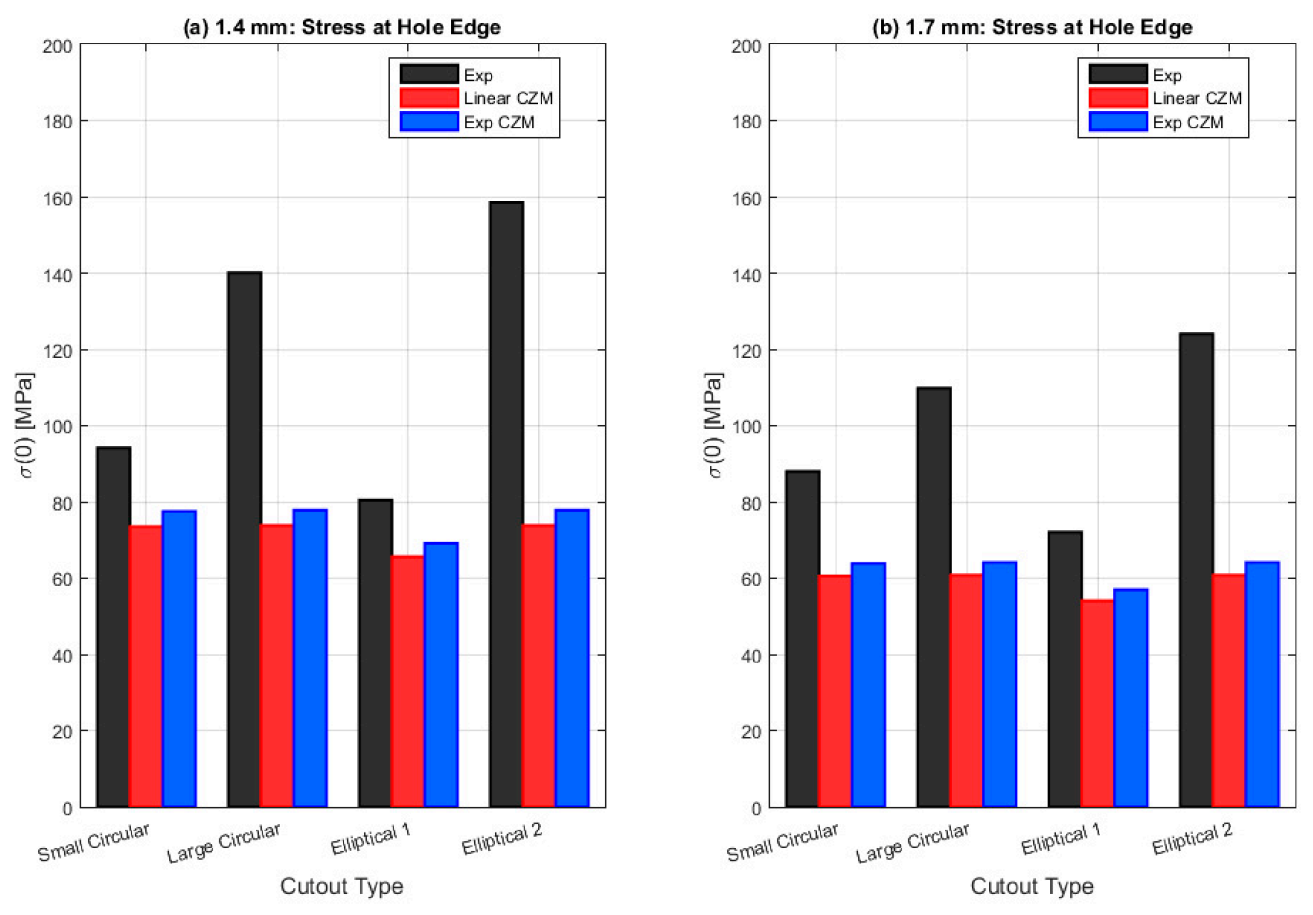
Comparison cutout types with absolute stress values.

The bar charts in Figure 8 provide a direct quantitative comparison of the absolute stress values concentrated at the immediate boundary of the cutout (*σ*(0) at *x* = 0) across the four studied geometries. Figure 8a and Figure 8b contrast the empirical findings with the analytical predictions of the linear and exponential cohesive zone models (CZMs) for the 1.4 mm and 1.7 mm laminate thicknesses, respectively. The experimental data (represented by the black bars) clearly demonstrate that the notch geometry and its orientation relative to the loading direction dictate the magnitude of the edge stress. The Elliptical 2 configuration (minor axis parallel to the load) introduces the most severe stress intensification, peaking at 158.49 MPa for the 1.4 mm laminate (Figure 8a) and 124.00 MPa for the 1.7 mm laminate (Figure 8b). In contrast, the Elliptical 1 configuration (major axis parallel to the load) proves to be the most structurally efficient, minimizing the localized edge stress to 80.41 MPa and 72.00 MPa for the respective thicknesses. Both analytical cohesive zone models systematically underpredict these localized edge stresses across all geometric configurations. While the experimental stress values vary significantly depending on the cutout type, the analytical predictions remain within a narrowly bounded range. Specifically, the linear CZM predicts edge stresses between 54 and 74 MPa, whereas the exponential CZM yields slightly higher values between 57 and 78 MPa. This uniform behavior occurs because the analytical framework relies heavily on the global material characteristic length scale (𝓁*_ch_*) and unnotched tensile properties, and consequently cannot fully capture the highly localized, geometry-dependent plastic deformation within the aluminum layers and micro-damage propagation along the polymer-composite interfaces at the notch perimeter. Despite these limitations, the exponential CZM (blue bars) consistently exhibits closer agreement with the experimental data than the linear softening law (red bars) across both laminate thicknesses, particularly evident in Figure 8a, where the stress concentrations are more severe, suggesting that the exponential traction–separation law offers a slight advantage in representing the complex near-tip damage mechanisms associated with aggressive notch geometries.

**Figure 9:**
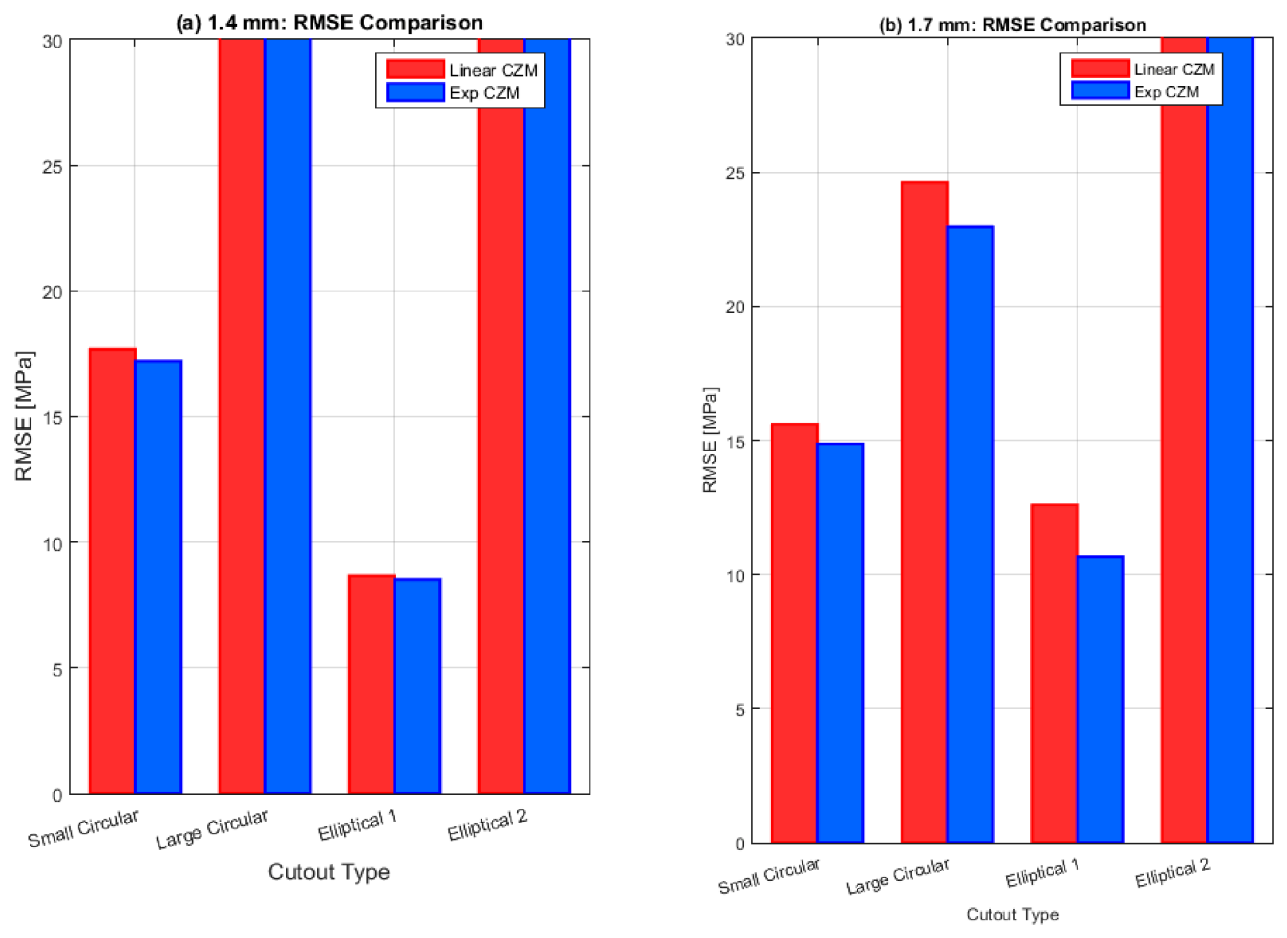
Comparison cutout types with RMSE.

Figure 9 presents the quantitative evaluation of the Root Mean Square Error (RMSE) values computed across the full ligament length for both the linear and exponential cohesive zone models. Figure 9a and Figure 9b contrast the statistical accuracy of the two formulations for the 1.4 mm and 1.7 mm laminate thicknesses, respectively. This metric evaluates the predictive capability of each traction–separation law over the entire stress field rather than evaluating a single boundary value.

The statistical results demonstrate that the exponential CZM consistently achieves superior predictive accuracy, yielding systematically lower RMSE values than the linear variant across all evaluated cutout shapes and plate thicknesses. For the 1.4 mm laminate (Figure 9a), the Elliptical 1 configuration (Major Axis||Load) registers the lowest error margins, falling below 10 MPa due to its smooth, well-distributed stress decay profile. In contrast, geometries with high structural bluntness perpendicular to the load trajectory—specifically the Large Circular and Elliptical 2 configurations—exhibit severe error growth, with RMSE values exceeding 25 to 30 MPa. This elevated error is fundamentally driven by the localized underpredictions near the notch perimeter (*x* = 0).

A critical comparison between the two plate thicknesses reveals a noticeable reduction in the average RMSE scores for the thicker 1.7 mm laminate (Figure 9b), particularly within the circular configurations. This improvement indicates that the analytical cohesive zone framework yields greater accuracy as the laminate thickness increases. The higher volume fraction of ductile aluminium 2024-T3 layers in the 1.7 mm laminate enhances plastic deformation capacity within the fracture process zone, aligning the physical stress redistribution mechanism more closely with the foundational continuum mechanics assumptions embedded in the cohesive softening laws. Overall, the exponential CZM demonstrates superior statistical performance across both thicknesses, with the most pronounced improvements observed for the thicker laminate.

It should be noted that the coefficient of determination (R^2^) reported in this study was computed against the pointwise stress values measured along the ligament (at *x* = 0, 17, 22, and 28 mm from the cutout edge), comparing the analytical predictions with the experimental stress data of Yazdani et al. [8]. The relatively low R^2^ values (0.281 and 0.311 for the 1.4 and 1.7 mm laminates, respectively) reflect the inherent difficulty of capturing the highly localized stress fields near the cutout edge using a global analytical framework. The primary sources of unexplained variance are: (i) the systematic underprediction of near-edge stresses at *x* = 0, where experimental values can be two to three times higher than model predictions due to localized plastic deformation in the aluminium layers and interfacial micro-damage; (ii) the use of a single characteristic length (*l_ch_*) and unnotched tensile properties, which cannot account for geometry-dependent damage mechanisms; (iii) the absence of explicit delamination and matrix cracking in the analytical formulation; and (iv) experimental scatter inherent to strain gauge measurements. Despite these limitations, the exponential CZM consistently achieves lower RMSE and higher R^2^ than the linear CZM across all cutout geometries and both laminate thicknesses, confirming its relative superiority for engineering predictions.

Addition, to properly contextualize the RMSE values, it is important to note that the experimental peak stresses at the cutout edge ranged from 72.00 MPa to 158.49 MPa for the 1.4 mm laminate and from 72.00 MPa to 124.00 MPa for the 1.7 mm laminate, depending on the cutout geometry (Figure 7 and Table 3 and Table 4). These peak stresses correspond to approximately 40–90% of the unnotched tensile strength (approximately 170–180 MPa). The reported RMSE values of 24.87 MPa (1.4 mm) and 20.99 MPa (1.7 mm) therefore represent approximately 15–25% of the maximum experimental stress. However, because the RMSE is computed across the entire ligament length, this value is dominated by the near-edge underprediction at *x* = 0; in the intermediate and far-field regions (*x* > 15 mm), the prediction errors are typically below 10%. Thus, the RMSE provides a conservative (upper-bound) estimate of the model error in the critical near-edge region, while the far-field stress decay is captured with significantly higher accuracy.

The improved prediction accuracy observed for the thicker 1.7 mm laminate is attributed to its higher aluminium volume fraction, which enhances the plastic deformation capacity within the fracture process zone and aligns more closely with the continuum assumptions embedded in the cohesive softening laws. While the experimental study of Yazdani et al. [8] did not report a detailed damage sequence analysis for the two thicknesses, it is reasonable to expect that the role of delamination and interlaminar shear may differ between the 1.4 mm and 1.7 mm laminates due to the different metal-to-composite ratio. The exponential CZM, with its smoother initial stiffness degradation, appears to better capture the more ductile stress redistribution in the thicker laminate. A definitive assessment of the dominant failure mode and damage sequence would require complementary experimental techniques such as acoustic emission monitoring, in situ microscopy, or fractographic analysis, which are recommended for future work.

**Figure 10:**
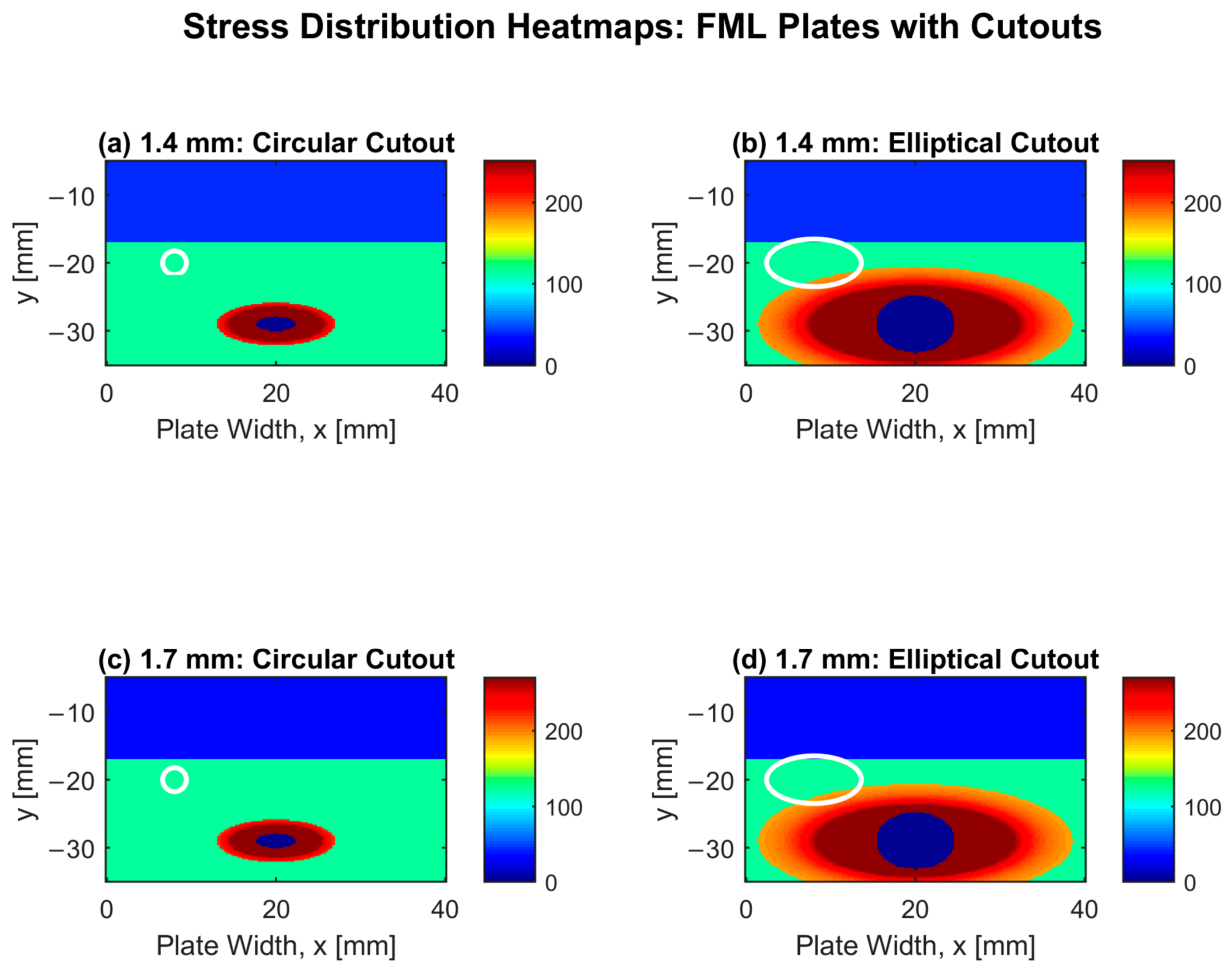
Stress distribution heatmaps: FML plates with cutouts.

**Figure 11:**
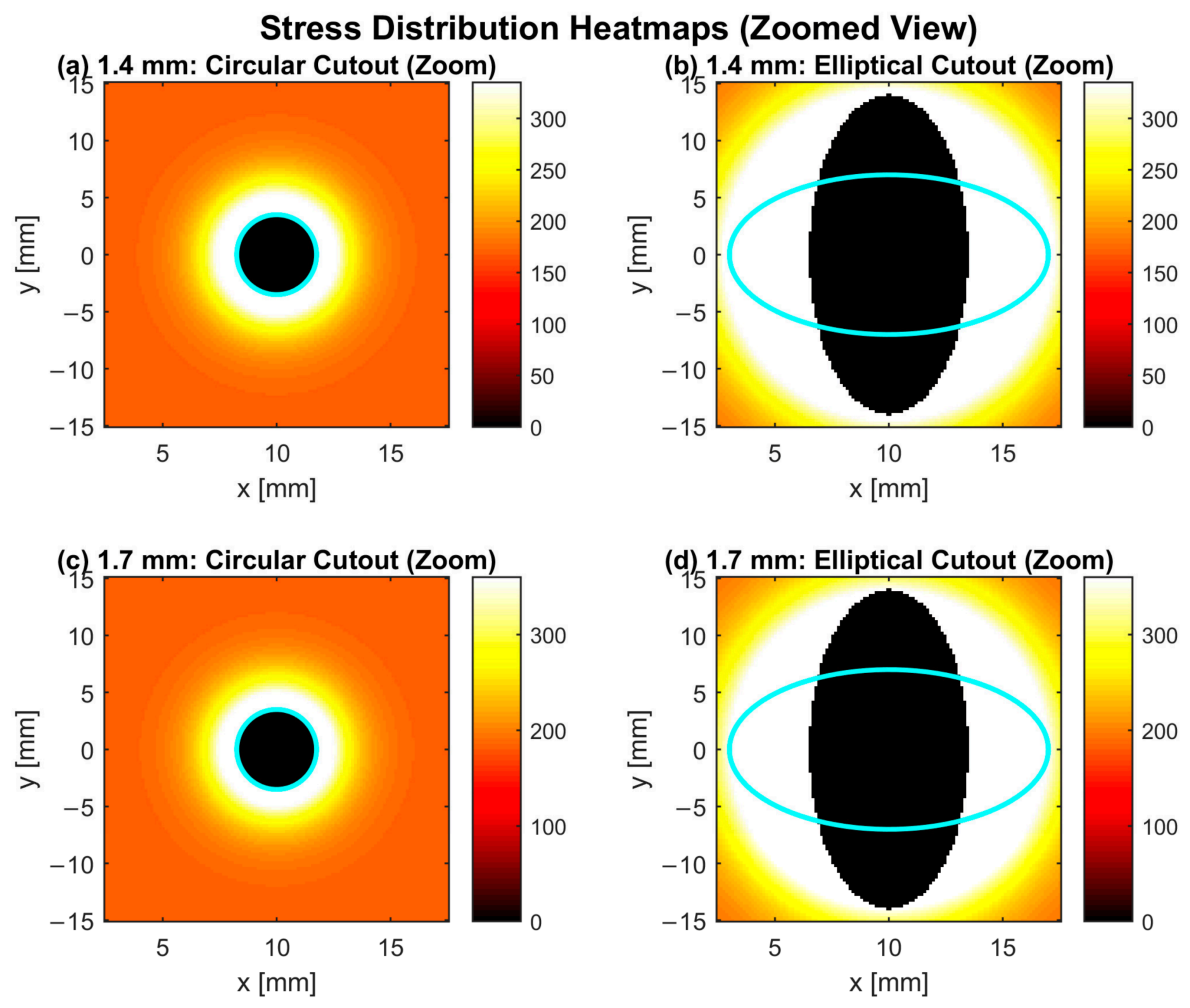
Stress distribution heatmaps (zoomed view): FML plates with cutouts.

**Figure 12:**
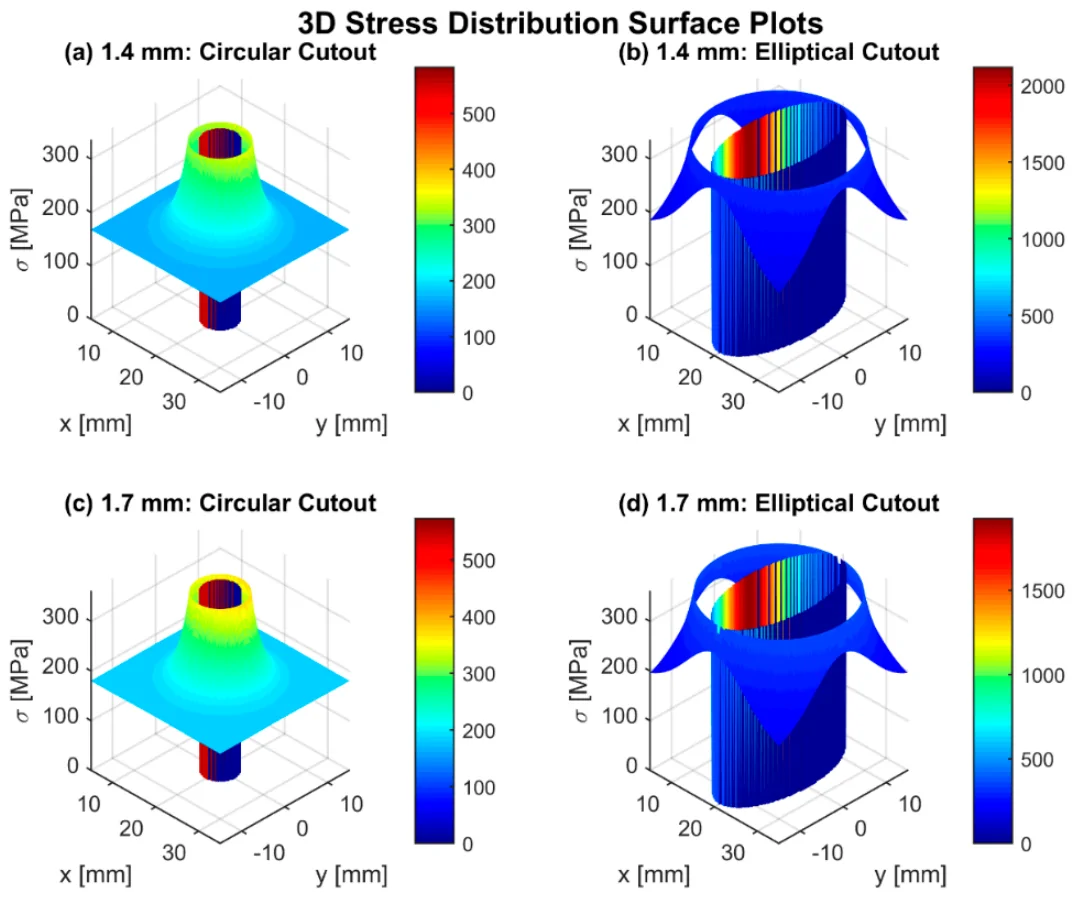
3-D stress distribution surfaces.

Figure 10, Figure 11 and Figure 12 provide a comprehensive, multi-dimensional visualization of the simulated stress fields across the perforated fiber–metal laminates (FMLs), mapping the global continuum response, high-resolution boundary zones, and topographic stress gradients, respectively.

The macro-scale heatmaps in Figure 10 clearly illustrate the spatial evolution of the stress trajectory under uniform tension. The material transitions from zero-stress zones (dark blue) inside the cutout boundaries through a stable far-field nominal plateau (green), reaching its highest concentration (deep red) symmetrically along the transverse ligament lines.

To isolate the extreme gradients driving damage initiation, the high-resolution close-up views in Figure 11 focus on the immediate notch perimeters. These views utilize a thermal colormap to reveal a localized stress rise approaching 300 MPa (white/yellow bands) right at the hole edge, which undergoes a rapid, non-linear decay within less than 5 mm of the bulk material. For the circular cutouts, this concentration forms an axisymmetric, ring-like band, whereas the elliptical cutouts present an elongated, highly concentrated oval perimeter where the severe localized fields are compressed tightly along the transverse boundaries.

This spatial decay behavior is quantitatively confirmed by the 3D topographic surface mappings in Figure 12. The circular configurations generate uniform, conical volcano-like stress hills, while the elliptical cutouts produce severe, ridge-like saddle topographies with steep vertical cliffs rising along the high-curvature apexes. Across all three visualization scales, the 1.4 mm laminates consistently show higher contour intensities and higher peak elevations than the 1.7 mm counterparts. This direct mechanical consequence stems from the reduced cross-sectional area carrying the applied load, validating how thickness and notch shape interact to alter the three-dimensional stress landscape and reinforcing the critical role of geometric design in mitigating stress concentration effects in FML structures.

## 5. Conclusions

The study comparatively evaluated linear and exponential cohesive zone models (CZMs) for predicting stress fields around cutouts in FML laminates of two thicknesses (1.4 and 1.7 mm). Both models successfully captured the macroscale stress decay along the ligament, though systematic underpredictions were observed at the immediate cutout boundary. The exponential CZM consistently outperformed the linear formulation, yielding lower RMSE values (24.87 and 20.99 MPa for the 1.4 and 1.7 mm laminates, respectively) and higher R^2^ values (0.281 and 0.311). The Elliptical 2 configuration produced the highest stress concentration factors (3.70 and 3.51), while Elliptical 1 yielded the lowest (1.88 and 2.04), confirming the structural benefit of aligning elliptical cutouts with the loading direction. Thicker laminates exhibited improved prediction accuracy due to increased aluminum content and enhanced plastic deformation capacity. The moderate R^2^ values reflect the challenges of capturing highly localized near-edge stress fields with a global analytical model; future work should incorporate geometry-dependent calibration of the characteristic length and explicit interfacial damage parameter. Despite near-edge limitations arising from the use of global unnotched parameters, the analytical CZM framework provides a computationally efficient alternative for engineering design. The exponential cohesive law is recommended for stress prediction in perforated FMLs, particularly for thicker laminates, with future work needed to incorporate localized damage mechanisms for improved near-tip accuracy.

The present analytical framework does not explicitly account for the damage sequence or the relative contribution of delamination versus plastic deformation, which may differ between laminate thicknesses. Future work should incorporate detailed damage characterization to further refine the cohesive law calibration for FMLs with varying metal-to-composite ratios. In addition, the analytical CZM runs in under a few seconds versus hours–days for equivalent 3D FE simulations (3–5 orders of magnitude faster), but is limited to single central cutouts under uniform tension without explicit delamination or matrix cracking, making it a rapid preliminary design tool rather than a replacement for detailed FE analysis.

## Figures and Tables

**Table 1:** Material properties of constituent layers reproduced from Ref. [8].

Material	E (GPa)	ν	Thickness (mm)
Al 2024-T3	71.1	0.33	0.3/0.4
Glass/Epoxy Woven	15.8	0.25	0.2

**Table 2:** Global mechanical properties of the FML laminates per Yazdani et al. [8].

Property	1.4 mm Laminate	1.7 mm Laminate
***E*_0_ (GPa)**	51.93	52.83
***E*_45_ (GPa)**	42.36	43.47
***G*_12_ (GPa)**	15.25	15.72
***ν*_12_**	0.25	0.25

**Table 3:** Experimental stress distribution data for the 1.4 mm laminate per Yazdani et al. [8].

Cutout Type	*x*_1_ (mm)	*σ*_1_ (MPa)	*x*_2_ (mm)	*σ*_2_ (MPa)	*x*_3_ (mm)	*σ*_3_ (MPa)	*x*_4_ (mm)	*σ*_4_ (MPa)
Small Circular	0	94.09	17.27	29.72	22.10	27.29	28.04	27.13
Large Circular	0	140.12	17.36	45.32	22.18	42.08	28.15	39.77
Elliptical 1	0	80.41	17.20	46.48	22.20	46.21	28.73	45.39
Elliptical 2	0	158.49	17.98	34.79	22.29	37.87	28.88	36.02

**Table 4:** Experimental stress distribution data for the 1.7 mm laminate per Yazdani et al. [8].

Cutout Type	*x*_1_ (mm)	*σ*_1_ (MPa)	*x*_2_ (mm)	*σ*_2_ (MPa)	*x*_3_ (mm)	*σ*_3_ (MPa)	*x*_4_ (mm)	*σ*_4_ (MPa)
Small Circular	0	88.00	18.00	27.00	20.00	25.50	22.00	26.00
Large Circular	0	109.80	17.00	46.27	22.00	38.43	28.00	36.08
Elliptical 1	0	72.00	17.20	50.00	20.00	51.00	25.00	53.00
Elliptical 2	0	124.00	17.20	32.10	28.00	31.00	—	—

**Table 5:** Experimental stress concentration factors calculated from the reported stress data in Yazdani et al. [8] using the stress concentration factor is defined as $\mathrm{SCF}=\frac{\sigma \left(x=0\right)}{\sigma_{\mathrm{nom}}}$.

Cutout Type	1.4 mm Laminate	1.7 mm Laminate
Small Circular	2.20	2.49
Large Circular	3.27	3.11
Elliptical 1	1.88	2.04
Elliptical 2	3.70	3.51

## Data Availability

The data supporting the findings of this study are available from the corresponding authors upon reasonable request.
